# *N*-Terminal Region of GbIspH1, *Ginkgo biloba* IspH Type 1, May Be Involved in the pH-Dependent Regulation of Enzyme Activity

**DOI:** 10.1155/2015/241479

**Published:** 2015-03-29

**Authors:** Bok-Kyu Shin, Joong-Hoon Ahn, Jaehong Han

**Affiliations:** ^1^Metalloenzyme Research Group and Department of Biotechnology, Chung-Ang University, Anseong 456-756, Republic of Korea; ^2^Department of Bioscience and Biotechnology, Bio/Molecular Informatics Center, Konkuk University, Seoul 143-701, Republic of Korea

## Abstract

GbIspH1, IspH type 1 in *Ginkgo biloba* chloroplast, is the Fe/S enzyme catalyzing the reductive dehydroxylation of HMBPP to isopentenyl diphosphate (IPP) and dimethylallyl diphosphate (DMAPP) at the final step of methylerythritol phosphate pathway in chloroplast. Compared to the bacterial IspH, plant IspH, including GbIspH1, has an additional polypeptide chain at the *N*-terminus. Here, biochemical function of the *N*-terminal region of GbIspH1 was investigated with the *N*-terminal truncated GbIspH1 (GbIspH1-truncated). Both wild type GbIspH1 (GbIspH1-full) and GbIspH1-truncated were catalytically active and produced IPP and DMAPP in a ratio of 15 : 1. Kinetic parameters of *K*_*M*_ (17.3 ± 1.9 and 14.9 ± 2.3 *µ*M) and *k*_cat_ (369 ± 10 and 347 ± 12 min^−1^) at pH 8.0 were obtained for GbIspH1-full and GbIspH1-truncated, respectively. Interestingly, GbIspH1-full and GbIspH1-truncated showed significantly different pH-dependent activities, and the maximum enzyme activities were obtained at pH 8.0 and 7.5, respectively. However, catalytic activation energies (*E*_*a*_) of GbIspH1-full and GbIspH1-truncated were almost the same with 36.5 ± 1.6 and 35.0 ± 1.9 kJ/mol, respectively. It was suggested that the *N*-terminal region of GbIspH1 is involved in the pH-dependent regulation of enzyme activity during photosynthesis.

## 1. Introduction

**Figure 1:**
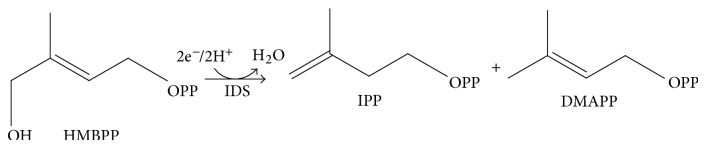


Since the discovery of mevalonate- (MVA-) independent methylerythritol phosphate (MEP) pathway [1], significant attentions have been drawn to the new isoprenoids biosynthetic pathway due to the potential applications in medicine [2]. Whereas MVA pathway is exclusively found in animals, Achaea, and fungi, MEP pathways are ubiquitous in most bacteria, protozoa, and cyanobacteria [3]. Interestingly, plant is known to utilize both pathways, as MVA and MEP pathways are found in cytosol and plastid, respectively. In MVA pathway, mevalonate produced from acetyl-CoA is converted to isopentenyl diphosphate (IPP) by three ATP-dependent enzymes. However, MEP pathway begins with the glycolysis metabolites, pyruvate and glyceraldehyde-3-phosphate, and seven unique enzymes comprise the pathway to produce IPP and dimethylallyl diphosphate (DMAPP) (see Figure 1).

In MEP pathway, 4-hydroxy-3-methylbut-2-enyl diphosphate (HMBPP) reductase (HDR, IspH, or LytB; EC 1.17.1.2) catalyzes the final step and the reductive dehydroxylation of HMBPP produces IPP and DMAPP [4, 5]. A 3 : 1 site-differentiated Fe_4_S_4_ cluster was identified at the active site of bacterial IspH by Mössbauer spectroscopy [6], and the protein X-ray crystallographic structures [7–9] provided the structural feature of the substrate binding site. At present, two different reaction mechanisms were proposed for IspH. Both mechanisms involve the same reaction steps of substrate binding, one electron reduction of the Fe/S cluster, and the electron transfer from the reduced [Fe_4_S_4_]^2+^ cluster to HMBPP. However, Birch reduction-like mechanism requires formation of the allylic radical or anionic intermediate [10, 11], whereas the organometallic mechanism proposes the *η* ^2^-alkenyl metallacycle intermediate formed between the Fe/S cluster and HMBPP [12].

Most mechanistic studies were carried out with the bacterial IspH [13], but no biochemical reports are available for plant IspH. Although plant IspH appears to follow the same reaction mechanism as bacterial IspH, its amino acid sequences are distinct from the bacterial IspH (see Figure S1 in Supplementary Material available online at http://dx.doi.org/10.1155/2015/241479). Namely, plant IspH proteins have extra 100–130 amino acid residues at the* N*-terminal region. To study the reaction mechanism of plant IspH, GbIspH1 (GbIspH1-full), IspH type 1 from* Ginkgo biloba*, was prepared. Besides, biochemical functions of the* N*-terminal region of the plant IspH were also investigated with the* N*-terminal truncated GbIspH1 (GbIspH1-truncated).

## 2. Methods

### 2.1. General Information

Methyl viologen (MV), sodium dithionite (DT), trizma base,* N*-cyclohexyl-2-aminoethanesulfonic acid (CHES), 4-(2-hydroxyethyl)-1-piperazineethanesulfonic acid (HEPES), 2-(*N*-morpholino)ethanesulfonic acid (MES), and disodium salt of 5,5′-[3-(2-pyridyl)-1,2,4-triazine-5,6-diyl]bis-2-furansulfonic acid (ferene) were purchased from Aldrich. For the anoxic environment (O_2_ < 1 ppm), Schlenk line and anaerobic chamber (M.O.Tech) equipped with Pd catalyst were employed. Enzyme substrate, HMBPP, was prepared according to the published method [14] and characterized by NMR spectroscope. CD spectra of proteins in the same buffer solution (5.71 *μ*M) were measured at the range of 190 nm to 260 nm with Chirascan plus (Applied Photophysics).

### 2.2. Expression and Purification of GbIspH1

GbIspH1 with His-tag (GbIspH1-full) was obtained from recombinant* Escherichia coli* BL21 (DE3) containing pQE-31 vector, which was kindly provided by Professor Soo-Un Kim at Seoul National University, Korea. The* N*-terminal polypeptide (Met1-Arg61) truncated GbIspH1 was subcloned into BamHI/NotI sites of pETDuet (Novagen). His6-tag was located at the* N*-terminal of both proteins, and GbIspH1-full and GbIspH1-truncated were purified using His-tag affinity column chromatography. The amino acid sequences of two proteins are available at Supplementary Material. The cell was grown in LB media containing 50 *μ*g/mL ampicillin, 1.0 mM FeCl_3_, and 1.0 mM (NH_4_)_2_SO_4_ until the OD_600_ reached 0.6 at 37°C. Cells were then incubated with 1.0 mM isopropyl-1-thio-*β*-D-galactopyranoside (IPTG) at 30°C for 13 hours to express the proteins. Cells were harvested by centrifugation (3,000 g, 15 min) at 4°C and stored at −70°C before use. Frozen cells (typically 10 g) were thawed at room temperature under nitrogen atmosphere and diluted with ten times volume of anaerobic lysis buffer (50 mM phosphate, pH 8.0, and 150 mM NaCl) on ice. Cell lysate was obtained from sonication (10 cycles of 20 sec burst with 1-minute break) under argon atmosphere. The supernatant was collected after centrifugation at 11,000 g for 20 minutes at 4°C. The supernatant was loaded on a Ni-NTA affinity column chromatography (4 mL resin) in anaerobic chamber. The resin was preequilibrated with the phosphate buffer (50 mM phosphate, pH 8.0, and 150 mM NaCl). Column was washed with phosphate buffer containing 20 mM imidazole and then eluted with phosphate buffer containing 200 mM imidazole. Fractions having a brown color were pooled and then desalted by Amicon ultra centrifugal filter (30 kDa) at 4000 g and the buffer exchanged to the Tris/HCl buffer (50 mM Tris/HCl, pH 8.0, and 100 mM NaCl). UV-Vis spectra of proteins are obtained from Scinco S-3150 UV-Vis spectrophotometer. Protein concentration was determined by Bradford assay [15]. The iron contents of the purified GbIspH1 proteins were determined by the absorption at 532 nm of Fe^2+^-ferene (disodium salt of 5,5′-[3-(2-pyridyl)-1,2,4-triazine-5,6-diyl]bis-2-furansulfonic acid) complex [16].

### 2.3. Enzyme Kinetics of GbIspH1

Enzyme kinetics study of GbIspH1-full and GbIspH1-truncated was carried out under strict anaerobic conditions. To the reaction mixture (2.0 mM MV, 0.4 mM DT, 50 mM HEPES, and pH 8.0) containing HMBPP, 200 nM of GbIspH1 was added to initiate the reaction. Total volume of enzyme reaction mixture was 450 *μ*L and the concentration of HMBPP was varied between 0 and 250 *μ*M for substrate-dependent kinetics. Reduction of HMBPP was monitored by the disappearance of reduced MV (*ε*_732_ = 3150 M^−1 ^cm^−1^) at 732 nm by UV-Vis spectrophotometer. Sigma plot software was used to obtain kinetics parameters from the initial reaction rates.

For pH-dependent kinetics study, GbIspH1 (200 nM) was added to the three-buffer reaction mixture (250 *μ*M HMBPP, 2.0 mM MV, 0.4 mM DT, 50 mM CHES, 50 mM HEPES, and 50 mM MES) after adjusting pH. Temperature-dependent kinetics carried out with 250 *μ*M of HMBPP at 0–50°C. GbIspH1 (200 nM) was added to the reaction mixture to initiate the reaction while the required reaction temperature was maintained.

### 2.4. Reaction Product Identification

To isolate reaction products, enzyme reaction was carried out with GbIspH1 (1 *μ*M), HMBPP (5.3 mM), MV (5.0 mM), and DT (7.5 mM) in 300 *μ*L of 50 mM HEPES buffer (pH 8.0) at room temperature for 3 h under anaerobic atmosphere. After reaction, cerium oxide (0.75 g) in 300 *μ*L 0.2 N ammonia solution was added to the enzyme reaction mixture [17]. The solution was reacted for 1 h at 40°C with mild stirring, and the dephosphorylated products were extracted with ethyl acetate (200 *μ*L × 3). For GC/MS analysis, Agilent 7890A (GC) equipped with DB-5MS (19091S-433) column (30 m, 0.25 mm, and 0.25 *μ*m) was connected with Agilent 5975C (MSD) detector. The sample (1 *μ*L) was injected to the injector (250°C) in splitless mode. Temperature of oven was kept at 40°C for 5 min before increasing to 450°C (10°C/min). Mass spectra were obtained with positive ionization mode by electron ionization (70 eV).

### 2.5. Sequence Analysis and Protein Structure Prediction

Amino acid sequences of IspH proteins were aligned using the MultAlin program (http://multalin.toulouse.inra.fr/multalin/multalin.html). Molecular weight and molar extinction coefficient of GbIspH1-full and GbIspH1-truncated were calculated using Protein Calculator v3.3 program (http://www.scripps.edu/~cdputnam/protcalc.html). Prediction of 3-dimensional protein structures of GbIspH1-full was achieved by I-TASSER (http://zhanglab.ccmb.med.umich.edu/I-TASSER) server.

## 3. Results and Discussion

Fe/S proteins participate in many important biological processes, such as photosynthesis and energy metabolism. Besides the electron transfer, Fe/S enzymes catalyze many unique reactions of nitrogen fixation, hydrogen production, CO oxidation, and more. Even simple cuboidal [4Fe_4_S] cluster acts as a cofactor in the radical SAM reaction and IspH reduction.

Whereas extensive biochemical characterization of bacterial IspH has been reported, no enzyme kinetics on plant IspH are available. Here, we first reported catalytic properties of GbIspH1. Kim et al. [18] suggested that the extra* N*-terminal peptide region contained the 10–40 residue-long transit peptides which may facilitate the translocation of IspH into chloroplast. However, biochemical function of the other* N*-terminal polypeptide region between the transit peptide and catalytic domain is not clear yet. The purpose of this study was to study the difference between plant IspH and bacterial IspH proteins and to investigate the possible function of the extra* N*-terminal polypeptide region of GbIspH1. The* N*-terminal truncated GbIspH1 used in this study was prepared by removing 61 amino acids at the* N*-terminus of GbIspH1.

### 3.1. Purification and Characterization of GbIspH1

GbIspH1 proteins of GbIspH1-full and GbIspH1-truncated were purified by Ni-NTA affinity column chromatography, and the purity of proteins was confirmed by SDS-PAGE (Figure S2a). The apparent molecular weights of GbIspH1-full and GbIspH1-truncated estimated from the band positions were 56.6 and 49.3 kDa, which were close to the theoretical values of 56.0 and 49.7 kDa, respectively. UV-Vis spectra of the purified GbIspH1-full and GbIspH1-truncated were obtained under anaerobic conditions, and the absorptions at 410 nm, *A*_410_, characteristic to the Fe_4_S_4_ cluster were observed for both (Figure S2b). The *A*_410_/*A*_280_ ratio of the [Fe_4_S_4_]^2+^ cluster-containing protein, measured by UV-Vis spectrophotometer, has been used to evaluate the percentage of holoprotein in the purified metalloprotein. The *A*_410_/*A*_280_ ratios of GbIspH1-full and GbIspH1-truncated were measured to be 0.18 and 0.17, respectively, and both proteins did not require reconstitution of the Fe_4_S_4_ cluster. Iron contents of GbIspH1-full and GbIspH1-truncated were also determined by colorimetric method using ferene, and 3.1 ± 0.2 and 3.5 ± 0.3 Fe/protein were determined for GbIspH1-full and GbIspH1-truncated, respectively. The incorporation of the Fe_4_S_4_ cluster in the purified GbIspH1 proteins was finally confirmed by EPR spectroscopy. The methyl viologen- (MV-) reduced WFT showed rhombic signals at 20 K characteristic to the reduced [Fe_4_S_4_]^+^ cluster (Figure S3). CD spectra obtained from GbIspH1-full and GbIspH1-truncated were very similar and indicated that both proteins were folded similarly (Figure S2c). The CD spectra were also similar to those reported from AaIspH [19], suggesting that IspH proteins from plant and bacteria are similar in their secondary structure compositions.

The purification of GbIspH1-full and GbIspH1-truncated proteins was carried out under oxygen-free conditions, and the identification of the Fe/S cluster required a few different biophysical characterizations. The isolated proteins exhibited pale brown color and the existence of the [Fe_4_S_4_]^2+^ cluster was evident from the electronic spectra. With protein quantitation and Fe analysis, incorporation of the [Fe_4_S_4_] cluster in GbIspH1-full and GbIspH1-truncated was confirmed. EPR spectroscopic study of the as-isolated and dithionite- (DT-) reduced proteins supported that the [Fe_4_S_4_] cluster in GbIspH1 shuttled +2 and +1 redox state.

### 3.2. Enzyme Kinetics of GbIspH1

To measure the enzyme activity of the purified IspH, electrons from the electron donor are required because IspH catalyzes the reductive dehydroxylation of HMBPP. A few* in vitro* electron donors, including biological NADPH/flavodoxin reductase/flavodoxin and abiological DT/MV reducing systems, have been utilized for the steady-state kinetics of IspH [11]. In this study, DT-reduced MV was used as an electron donor and substrate-dependent reaction rates of GbIspH1-full and GbIspH1-truncated were measured in the presence of HMBPP by spectrophotometry. In detail, one-electron reduced MV exhibits two strong electronic absorptions at 732 nm (*ε*_732_ = 3150 M^−1 ^cm^−1^) and 604 nm (*ε*_604_ = 18750 M^−1 ^cm^−1^), and two molecules of reduced MV are consumed for the product formation by IspH. The electron transfer and HMBPP reduction are tightly coupled in the IspH catalysis, and the oxidation of the reduced MV monitored by the electronic absorption decrease is related to the IspH activity [20].

**Figure 2:**
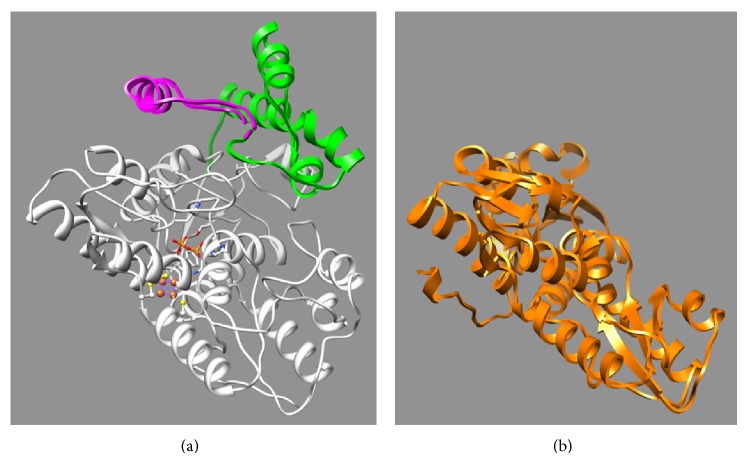
I-TASSER predicted structure of GbIspH1-full (a) is presented with bacterial IspH ((b), 3KE8 in orange). Residues of the conserved Cys, His, and Phe are depicted with ball and stick model, and the substrate HMBPP is represented with stick model. The extra* N*-terminal regions (magenta: 1–61, green: 52–131) were colored differently.

Substrate-dependent initial velocity curves of GbIspH1-full and GbIspH1-truncated showed typical Michealis-Menten kinetics (Figure S4), and catalytic activities of both GbIspH1 enzymes were comparable to the other reported IspH proteins (Table 1). For example, IspH from* E. coli* with the same reducing system showed *k*_cat_ 604 min^−1^ and *K*_*M*_ 19.7 *μ*M [21]. Between GbIspH1-full and GbIspH1-truncated, similar kinetic parameters of *k*_cat_ (369 ± 10 versus 347 ± 12 min^−1^) and *K*_*M*_ (17.3 ± 1.9 versus 14.9 ± 2.3 *μ*M) were obtained, respectively. Although it is not distinctively different, GbIspH1-full appears to exhibit better *k*_cat_ and higher *K*_*M*_. In the predicted three-dimensional protein structure of GbIspH1,* N*-terminal peptide is located near the substrate binding site (Figure 2). The relatively low *K*_*M*_ value obtained from GbIspH1-truncated may be due to the absence of extra* N*-terminal peptide chain which is presumably positioned on top of the substrate binding site.

**Figure 3:**
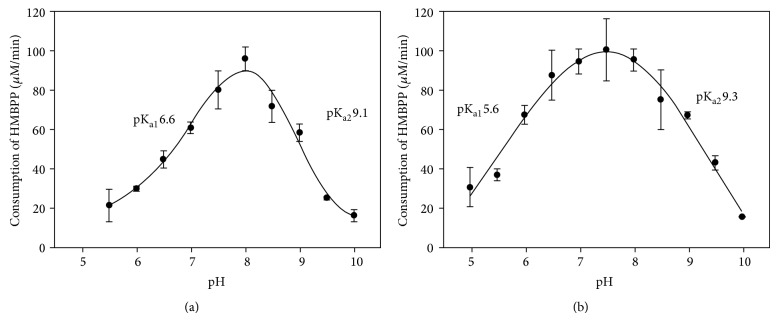
The pH-dependent activity profiles of GbIspH1-full (a) and GbIspH1-truncated (b).

Activity profiles of GbIspH1-full and GbIspH1-truncated at the range of pH 5.5–10 showed that two GbIspH1 proteins exhibited maximum activity at different pH values. Whereas GbIspH1-full showed the maximum enzyme activity at pH 8.0, GbIspH1-truncated showed the highest activity at pH 7.5. Altincicek et al. [22] reported that the maximum activity of IspH from* Aquifex aeolicus* was found in the range of pH 7.0–7.5. When the pH range of more than 50% maximum activity was compared, the range of GbIspH1-truncated (pH 5.6–9.3) was much broader than that of GbIspH1-full (pH 6.6–9.1) (Figure 3). Acidic pH region showing 50% maximum activity especially was shifted by 1.0 pH unit in GbIspH1-full. Therefore, it was proposed that extra* N*-terminal region of GbIspH1-full influenced the pH dependency of enzyme activity. Certain amino acid residues in the* N*-terminal region may modulate the proton transfer steps which are required during the catalysis, or conformational changes accompanied during the IspH catalysis could be perturbed in case of GbIspH1-truncated by truncating the* N*-terminal region of GbIspH1-full.

**Figure 4:**
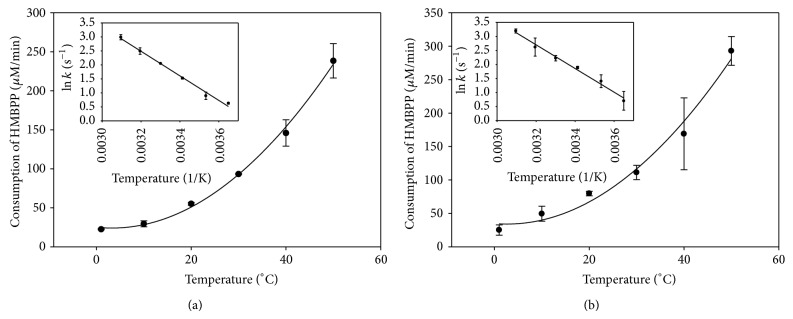
Temperature-dependent activity profiles of GbIspH1-full (a) and GbIspH1-truncated (b) with the Arrhenius plot inserts.

To find whether the extra* N*-terminal peptide region of GbIspH1 influences the enzyme reaction pathway, activation energy values of GbIspH1-full and GbIspH1-truncated were determined by measuring temperature-dependent enzyme activities at 0–50°C. As shown in Figure 4, temperature-dependent reaction rate constants of both enzymes were almost identical and the activation energy (*E*_*a*_) values obtained by Arrhenius equation were 36.5 ± 1.6 and 35.0 ± 1.9 kJ/mol for GbIspH1-full and GbIspH1-truncated, respectively. If the extra* N*-terminal peptide region of GbIspH1-full affected rate-limiting reaction step, the activation energy values of the reactions catalyzed by GbIspH1-full and GbIspH1-truncated are expected to be significantly different. However, the result strongly suggested that the extra* N*-terminal region did not alter the rate-limiting step of GbIspH1 catalysis. The activation energy values obtained from GbIspH1-full and GbIspH1-truncated were much higher than the reported *E*_*a*_ 8.0 kJ/mol of* Plasmodium falciparum* IspH [23] and comparable to the activation energy of 49 ± 2 kJ/mol reported for the thermophilic* A. aeolicus* [22].

Although it is not known yet which reaction step in the IspH enzyme reaction pathway is the rate-limiting step, Xiao et al. [21] reported that the activity of IspH could be significantly increased by changing electron donors. Furthermore, the conformational change following the electron transfer step is generally found as the rate-limiting step of the redox-active metalloenzyme catalysis. For example, conformational change of MoFe protein required to separate docked Fe protein after electron transfer is the rate-limiting step of nitrogenase catalysis. Therefore, it is very likely that the reaction step involving separation of the biological electron donor from IspH would be rate-limiting step. Based on the activation energy comparison, it is also proposed that the* N*-terminal region of GbIspH1 does not actively participate in the electron transfer step.

### 3.3. Reaction Products Identification

In plant cell, both MVA pathway and MEP pathway are found in cytosol and chloroplast, respectively, and believed to have different physiological functions [24]. It is also known that MVA pathway produces only IPP but MEP pathway provides IPP and DMAPP. Different ratio of IPP/DMAPP is required for the specific isoprenyl diphosphate biosynthesis in cytosol and chloroplast, and apparently more IPP than DMAPP is required for the synthesis of geranyl diphosphate (GPP), farnesyl diphosphate (FPP), and so on. Even though short chain prenyl diphosphate, including IPP (C5), DMAPP (C5), GPP (C10), and FPP (C15), can be transported to other cell organelles in plant cell [25], isoprenyl diphosphate isomerase (IDI) is universally found from the cell organelles including chloroplast, mitochondria, and peroxisome. Hence, maintaining the optimum metabolic flux of IPP/DMAPP in the cell looks critical [26]. This background information motivated us to study the products ratio of IPP/DMAPP produced by IspH.

**Figure 5:**
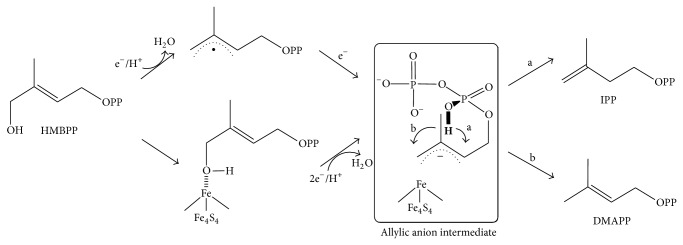
Proposed IspH-catalyzed reaction mechanism of isopentenyl diphosphate biosynthesis. Steps a and b represent possible proton transfer pathways to the allylic anion intermediate, which results in different product formation.

Interestingly, the ratio of IPP/DMAPP was also recognized as an important trait related with the reaction mechanism. According to the proposed IspH-catalyzed reaction mechanisms, the allylic anion species is commonly formed by two different IspH reaction mechanism, either from the reduction of substrate allylic radical species [7] or from the organometallic complex formation between substrate and the Fe_4_S_4_ cluster [8] (Figure 5). The allylic anion species was suggested as the key intermediate by both mechanisms that controls product formation and therefore determines the products ratio. Proton transfer from the intermediate diphosphate group to the allylic anion moiety is the key reaction step to control the product distribution of IPP and DMAPP. So far, all IspH proteins, including* P. falciparum* IspH, were reported to produce 4.5–6.3 : 1 ratio of IPP/DMAPP [27]. Recently, we have reported that the IDI-missing plant pathogenic bacterium* Burkholderia glumae* produced 2 : 1 ratio of IPP and DMAPP [28].

Identification of IPP and DMAPP produced by GbIspH1-full and GbIspH1-truncated was achieved by GC/MS analysis of the dephosphorylated products of IPP and DMAPP (Figure S5), and the products ratio was obtained by GC-FID quantitation (Supplementary Material). Controlled experiment with mixtures of standard IPP and DMAPP found that the recovery efficiency of prenyl and isoprenyl alcohols was 1 : 0.92, and the experimental results were corrected accordingly. Surprisingly, the ratios of IPP/DMAPP from the reaction mixtures of GbIspH1-full and GbIspH1-truncated were both found as 15 : 1 reproducibly. Because the products ratios obtained from GbIspH1 proteins were significantly larger than the reported values from other IspH proteins, it was proposed that the proton transfer to the allylic anion intermediate during the GbIspH1 catalysis could be different from the bacterial IspH system.

## 4. Conclusions

In summary, catalytically active GbIspH1 proteins of GbIspH1-full and GbIspH1-truncated were successfully purified and characterized under anaerobic conditions. The isolated GbIspH1 proteins contained the Fe_4_S_4_ cluster like bacterial IspH, and it showed comparable catalytic activity to the other IspH proteins. The extra* N*-terminal region of GbIspH1, common to plant IspH proteins, appears not to influence the rate-limiting step, because GbIspH1-full and GbIspH1-truncated showed similar kinetic parameters of *k*_cat_ and activation energy. However, the extra* N*-terminal region was suggested to modulate the enzyme activity in a pH-dependent manner, based on the pH-dependent activity profiles of GbIspH1-full and GbIspH1-truncated.

GbIspH1-full with complete GbIspH1 polypeptide was measured to have a maximum enzyme activity at pH 8.0, and IspH from* A. aeolicus* was reported to show the maximum enzyme activity in the range of pH 7.0–7.5 [22]. Interestingly, spinach IspG, another plant MEP pathway enzyme, found in chloroplast was reported to accept electrons from photosynthetic light reaction [29], and the expression of GbIspH1 was enhanced by light triggering [18]. With all of these reports, it is likely that photosynthetic regulation is operating in plant MEP pathway. During the photosynthetic light reaction, proton is transported from chloroplast stroma to thylakoid lumen, and the pH value of stroma approaches pH 8.0 [30]. From our pH-dependent activity study, it is plausible that the* N*-terminal polypeptide region of GbIspH1 could modulate the enzyme activity in a pH-dependent manner in chloroplast.

## Supplementary Material

Supplementary Material containing experimental details and more supporting data.

## Figures and Tables

**Table 1:** Kinetic parameters of IspH proteins.

Source	Reducing system	*A*_410_/*A*_280_	Kinetics parameters				Reference
			*k*_cat_ (min^−1^)^b^	S.A. (*μ*molmin^−1^mg^−1^)	*K*_*M*_ (*μ*M)	*k*_cat_/*K*_*M*_ (*µ*M^−1^ min^−1^)	
*E. coli *	NADPH^a^	0.4	11.6	0.31	15	0.8	[11]
	DT-MV	0.4	604	16.3	19.7	30.7	[21]
	DT-MDQ^c^	0.4	1125	30.4	31.6	35.6	

*A. aeolicus *	DT-MV		222	6.6	590	0.4	[22]

*P. falciparum *	DT-MV		124	2.1	39	3.2	[23]
	NADPH			10.3			

GbIspH1-full	DT-MV	0.17	369	6.7	17.3	21.3	This work
GbIspH1-truncated		0.18	347	7.2	14.9	23.3	

^a^NADPH was added with flavodoxin/flavodoxin reductase or ferredoxin/ferredoxin-NADP^+^ reductase.

^b^MW was estimated based on the peptide sequence. IspH from *E.  coli*, 37.0 kDa; *A. aeolicus*, 31.8 kDa; *P.  falciparum*, 58.8 kDa.

^c^6,7-dihydro-2,11-dimethyldipyrido[1,2-*a*:2,1-*c *]pyrazinium dibromide (MDQ) is a MV analog.

## References

[1] Rohmer M., Knani M., Simonin P., Sutter B., Sahm H. (1993). Isoprenoid biosynthesis in bacteria: a novel pathway for the early steps leading to isopentenyl diphosphate. *Biochemical Journal*.

[2] Kuzuyama T., Seto H. (2012). Two distinct pathways for essential metabolic precursors for isoprenoid biosynthesis. *Proceedings of the Japan Academy, Series B: Physical and Biological Sciences*.

[3] Eisenreich W., Bacher A., Arigoni D., Rohdich F. (2004). Biosynthesis of isoprenoids via the non-mevalonate pathway. *Cellular and Molecular Life Sciences*.

[4] Adam P., Hecht S., Eisenreich W. (2002). Biosynthesis of terpenes: studies on 1-hydroxy-2-methyl-2-(E)-butenyl 4-diphosphate reductase. *Proceedings of the National Academy of Sciences of the United States of America*.

[5] Rohdich F., Hecht S., Bacher A., Eisenreich W. (2003). Deoxyxylulose phosphate pathway of isoprenoid biosynthesis. Discovery and function of ispDEFGH genes and their cognate enzymes. *Pure and Applied Chemistry*.

[6] Seemann M., Janthawornpong K., Schweizer J. (2009). Isoprenoid biosynthesis via the MEP pathway: *in vivo* Mössbauer spectroscopy identifies a [4Fe-4S]^2+^ center with unusual coordination sphere in the LytB protein. *Journal of the American Chemical Society*.

[7] Gräwert T., Rohdich F., Span L. (2009). Structure of active IspH enzyme from escherichia coli provides mechanistic insights into substrate reduction. *Angewandte Chemie International Edition*.

[8] Gräwert T., Span I., Eisenreich W. (2010). Probing the reaction mechanism of IspH protein by X-ray structure analysis. *Proceedings of the National Academy of Sciences of the United States of America*.

[9] Rekittke I., Wiesner J., Röhrich R. (2008). Structure of (*E*)-4-hydroxy-3-methyl-but-2-enyl diphosphate reductase, the terminal enzyme of the non-mevalonate pathway. *Journal of the American Chemical Society*.

[10] Rohdich F., Zepeck F., Adam P. (2003). The deoxyxylulose phosphate pathway of isoprenoid biosynthesis: studies on the mechanisms of the reactions catalyzed by IspG and IspH protein. *Proceedings of the National Academy of Sciences of the United States of America*.

[11] Xiao Y., Zhao Z. K., Liu P. (2008). Mechanistic studies of IspH in the deoxyxylulose phosphate pathway: heterolytic C-O bond cleavage at C4 position. *Journal of the American Chemical Society*.

[12] Wang W., Wang K., Liu Y.-L. (2010). Bioorganometallic mechanism of action, and inhibition, of IspH. *Proceedings of the National Academy of Sciences of the United States of America*.

[13] Span I., Wang K., Wang W. (2012). Discovery of acetylene hydratase activity of the iron-sulphur protein IspH. *Nature Communications*.

[14] Hecht S., Amslinger S., Jauch J. (2002). Studies on the non-mevalonate isoprenoid biosynthetic pathway. Simple methods for preparation of isotope-labeled (*E*)-1-hydroxy-2-methylbut-2-enyl 4-diphosphate. *Tetrahedron Letters*.

[15] Bailey M. J. A., Noble J. W. (2009). Quantitation of protein. *Methods in Enzymology*.

[16] Kennedy M. C., Kent T. A., Emptage M., Merkle H., Beinert H., Münck E. (1984). Evidence for the formation of a linear [3Fe-4S] cluster in partially unfolded aconitase. *Journal of Biological Chemistry*.

[17] Tan F., Zhang Y., Wang J., Wei J., Cai Y., Qian X. (2008). An efficient method for dephosphorylation of phosphopeptides by cerium oxide. *Journal of Mass Spectrometry*.

[18] Kim S.-M., Kuzuyama T., Kobayashi A., Sando T., Chang Y.-J., Kim S.-U. (2008). 1-Hydroxy-2-methyl-2-(*E*)-butenyl 4-diphosphate reductase (IDS) is encoded by multicopy genes in gymnosperms *Ginkgo biloba* and *Pinus taeda*. *Planta*.

[19] Xu W., Lees N. S., Hall D., Welideniya D., Hoffman B. M., Duin E. C. (2012). A closer look at the spectroscopic properties of possible reaction intermediates in wild-type and mutant (*E*)-4-hydroxy-3-methylbut-2-enyl diphosphate reductase. *Biochemistry*.

[20] Kim H. S., Shin B.-K., Han J. (2014). High-throughput HDR inhibitor screening. *Journal of the Korean Society for Applied Biological Chemistry*.

[21] Xiao Y., Chu L., Sanakis Y., Liu P. (2009). Revisiting the IspH catalytic system in the deoxyxylulose phosphate pathway: achieving high activity. *Journal of the American Chemical Society*.

[22] Altincicek B., Duin E. C., Reichenberg A. (2002). LytB protein catalyzes the terminal step of the 2-C-methyl-D-erythritol-4-phosphate pathway of isoprenoid biosynthesis. *FEBS Letters*.

[23] Röhrich R. C., Englert N., Troschke K. (2005). Reconstitution of an apicoplast-localised electron transfer pathway involved in the isoprenoid biosynthesis of *Plasmodium falciparum*. *FEBS Letters*.

[24] Rodríguez-Concepción M. (2006). Early steps in isoprenoid biosynthesis: multilevel regulation of the supply of common precursors in plant cells. *Phytochemistry Reviews*.

[25] Bick J. A., Lange B. M. (2003). Metabolic cross talk between cytosolic and plastidial pathways of isoprenoid biosynthesis: unidirectional transport of intermediates across the chloroplast envelope membrane. *Archives of Biochemistry and Biophysics*.

[26] Pulido P., Perello C., Rodríguez-Concepción M. (2012). New insights into plant isoprenoid metabolism. *Molecular Plant*.

[27] Gräwert T., Kaiser J., Zepeck F. (2004). IspH protein of *Escherichia coli*: studies on iron-sulfur cluster implementation and catalysis. *Journal of the American Chemical Society*.

[28] Kwon M., Shin B.-K., Lee J., Han J., Kim S.-U. (2013). Characterization of *Burkholderia glumae* BGR1 4-hydroxy-3-methylbut-2-enyl diphosphate reductase (HDR), the terminal enzyme in 2-*C*-methyl-d-erythritol 4-phosphate (MEP) pathway. *Journal of the Korean Society for Applied Biological Chemistry*.

[29] Seemann M., Tse Sum Bui B., Wolff M., Miginiac-Maslow M., Rohmer M. (2006). Isoprenoid biosynthesis in plant chloroplasts via the MEP pathway: direct thylakoid/ferredoxin-dependent photoreduction of GcpE/IspG. *FEBS Letters*.

[30] Hauser M., Eichelmann H., Oja V., Heber U., Laisk A. (1995). Stimulation by light of rapid pH regulation in the chloroplast stroma in vivo as indicated by CO_2_ solubilization in leaves. *Plant Physiology*.

