# Supplementary material for: N-Terminal Region of GbIspH1, Ginkgo biloba IspH Type 1, May Be Involved in the pH-Dependent Regulation of Enzyme Activity
Source: Bioinorg Chem Appl. 2015 Mar 29;2015:241479. doi: 10.1155/2015/241479 (PMC4393896; doi:10.1155/2015/241479)
Supplement: Supplementary file 1 — Supplementary Material containing experimental details and more supporting data. [file 241479.f1.docx]

Supplementary Material

***N*-Terminal Region of GbHDR1, *Ginkgo biloba* 4-Hydroxy-3-methylbut-2-enyl Diphosphate Reductase Type 1, May Be Involved in the pH-Dependent Regulation of Enzyme Activity**

Bok-Kyu Shin,^1^ Joong-Hoon Ahn^2^ and Jaehong Han^1^*

**Experimental details**

**E1. Fe Analysis**^1,2^

The iron content of GbHDR1 was measured by absorption at 593 nm of Fe^2+^-ferene (disodium salt of 5,5'-[3-(2-pyridyl)-1,2,4-triazine-5,6-diyl]bis-2-furansulfonic acid) complex. Three solutions, **a**, **b**, and **c**, were prepared before the measurement. **a**; sodium dodecyl sulfate (1.35 g, 4.68 mmol) in 30 mL of H_2_O was mixed with 0.45 mL of saturated sodium acetate solution, **b**; ascorbic acid (270 mg, 1.50 mmol) and sodium metabisulfite (Na_2_S_2_O_5_, 9 mg, 0.05 mmol) in 5.6 mL of H_2_O was mixed with 0.4 mL of saturated sodium acetate solution, and **c**; ferene (18 mg, 0.036 mmol) in 1 mL of H_2_O. Protein solution (50 µL) was mixed with 50 µL of solution **a**, and then followed by addition of solution **b** (50 µL). After incubation at 30 ºC for 15 minutes, 2.5 µL of solution **c** was added to the mixture. The final solution was stored at room temperature at least for 5 min before the detection at 593 nm. A calibration curve was obtained with iron(II) perchlorate at the range of 0 – 100 µM of Fe ions. Calibration curve; y = 0.0075 x + 0.0395, R^2^ = 0.9905.


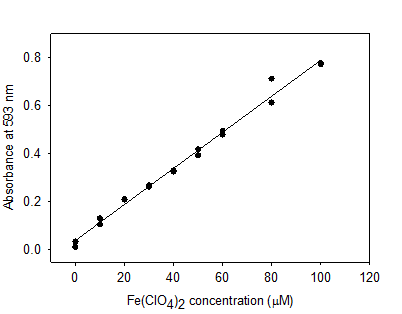


| GbHDR1 | Fe contents (μM) | | | Protein (μM) | | | Fe/protein | | |
| --- | --- | --- | --- | --- | --- | --- | --- | --- | --- |
|  | AVE | SD | RSD | AVE | SD | RSD | AVE | SD | RSD |
| **GbHDR1-full** | **1,059.1** | **29.7** | **2.8** | **343** | **26** | **7.6** | **3.09** | **0.25** | **8.1** |
| **GbHDR1-truncated** | **1,293.7** | **51.5** | **4.0** | **371** | **23** | **6.2** | **3.49** | **0.26** | **7.4** |

1. Kennedy, M. C., Kent, T. A., Emptage, M., Merkle, H., Beinert, H., and Munck, E. (1984) Evidence for the formation of a linear [3Fe-4S] cluster in partially unfolded aconitase. J Biol Chem 259, 14463-14471.
2. Yan, A., and Kiley, P. J. (2009) Techniques to isolate O2-sensitive proteins: [4Fe-4S]-FNR as an example. Methods Enzymol 463, 787-805.**E2. Products Recovery Test**

Dephosphorylation of IPP and DMAPP

Under the same enzyme reaction conditions, (NH_4_)_3_IPP and (NH_4_)_3_DMAPP (each 168 μM) were added to the HEPES buffer solution (50 mM, pH 8.0, 150 μL) containing GbHDR1-full (1.5 μM), MV (5.0 mM), and DT (7.5 mM). To this, 10 μL of ammonia solution (1.6 N) and 0.1 g of cerium oxide powder were added at 40 °C and reacted for 2 hrs with stirring. The reaction mixture was centrifuged at 10770 *g* for 5 minutes and extracted with 170 μL of ethyl acetate.

Standard curve of products

The alcohol products were analyzed by gas chromatography (GC) equipped with Agilent HP-5 capillary column and flame-ionization detector (FID). The inlet temperature was 250 °C and detector temperature was 300 °C. After injection, the oven temperature was maintained at 40 °C for 5 minutes, and raised to 150 °C with a 10 °C/min rate.

Standard curves of 3-methyl-3-buten-1-ol and 3-mthyl-2-buten-1-ol were obtained at the concentrations of 50 – 800 μL and at 10 – 100 μL, respectively. The linear relationship between peak integration and concentration were obtained with the equations of y (peak area) = 10,490,000 (± 126,000) x (mM) + 31,000 (± 52,000) (*R*^2^ = 0.9996) and y = 10,515,000 (± 203,000) x – 74,000 (± 12,000) (*R*^2^ = 0.9993) for 3-methyl-3-buten-1-ol and 3-mthyl-2-buten-1-ol, respectively.

Results

The GC peaks of 3-methyl-3-buten-1-ol and 3-mthyl-2-buten-1-ol (hydrolyzed alcohol products of IPP and DMAPP, respectively) were detected at 7.12 ± 0.02 and 8.17 ± 0.02 minutes, respectively. The average recovery yields of the alcohols were 77.4% and 69.7%, and the recovery ratio was 1 : 0.92 for 3-methyl-3-buten-1-ol : 3-mthyl-2-buten-1-ol.

GC chromatogram of standard compounds. Broken line shows control injection.

Standard curves of 3-methyl-3-buten-1-ol (left) and 3-mthyl-2-buten-1-ol (right).

| **Inj** | **3M3B1ol** | | | **3M2B1ol** | | |
| --- | --- | --- | --- | --- | --- | --- |
|  | **RT** | **peak area** | **mM** | **RT** | **peak area** | **mM** |
| 1_1 | 7.13 | 1319942 | 0.123 | 8.19 | 1099189 | 0.112 |
| 1_2 | 7.12 | 1324121 | 0.123 | 8.18 | 1128518 | 0.114 |
| 2_1 | 7.07 | 1383862 | 0.129 | 8.14 | 1160258 | 0.117 |
| 2_2 | 7.13 | 1499265 | 0.140 | 8.19 | 1248426 | 0.126 |
| 3_1 | 7.11 | 1400985 | 0.131 | 8.17 | 1131915 | 0.115 |
| 3_2 | 7.13 | 1438438 | 0.134 | 8.18 | 1169034 | 0.118 |
| **AVE** | 7.12 | 1394435 | 0.130 | 8.17 | 1156223 | 0.117 |
| **SD** | 0.02 | 68663.59 | 0.007 | 0.02 | 51561.8 | 0.005 |

| **Compound** | **RT** | **Area** | **µM** | **Recovery (%)*** |
| --- | --- | --- | --- | --- |
| 3M3B1ol | 7.12 ± 0.02 | 1394435 ± 72971 | 130 ± 7 | 77.4 |
| 3M2B1ol | 8.17 ± 0.02 | 1156223 ± 51562 | 117 ± 5 | 69.7 |
| * Calcd from 0.168 mM. | | |  | |

**Figure S1**. Amino acid alignment of HDRs from *Ginkgo biloba* (GbIDS1), *Pinus taeda* (PtIDS1), *Arabidopsis thaliana* (AtIDS), *Ostreococcus lucimarinus* (OlIDS), *Escherichia coli* (EcISpH), and *Aquifex aeolicus* (AaIspH). Conserved amino acid residues mentioned in the text were marked with triangles.


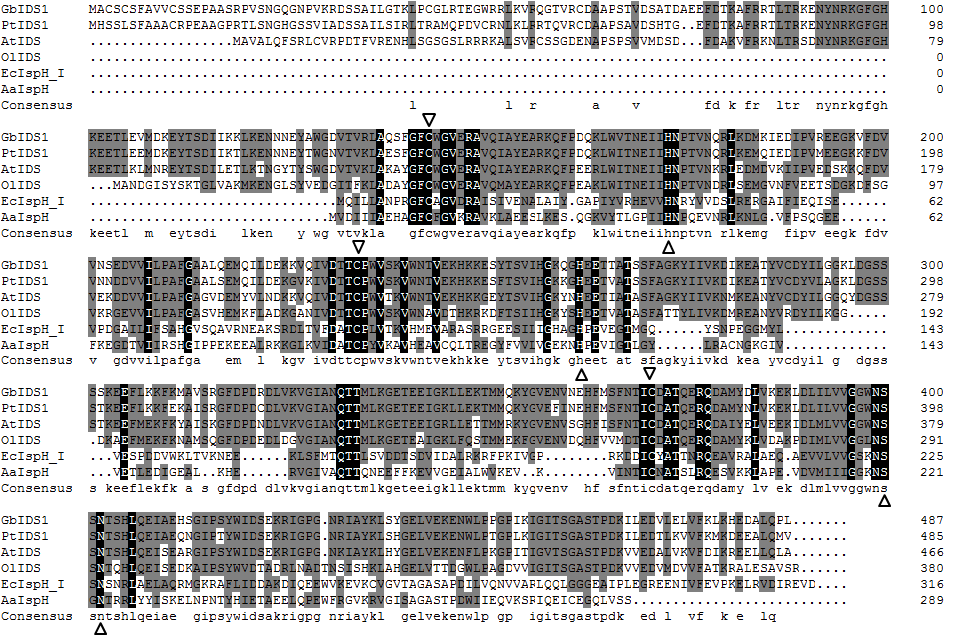


**Figure S2**. (A) SDA-PAGE of GbHDR1-full (left) and GbHDR1-truncated (right). Size marker (SM), crude extract (CE), flow through (FT) and purified proteins (P) were represented. (B) UV-Vis and (C) CD spectra of GbHDR1-full (solid line) and GbHDR1-truncated (broken line).


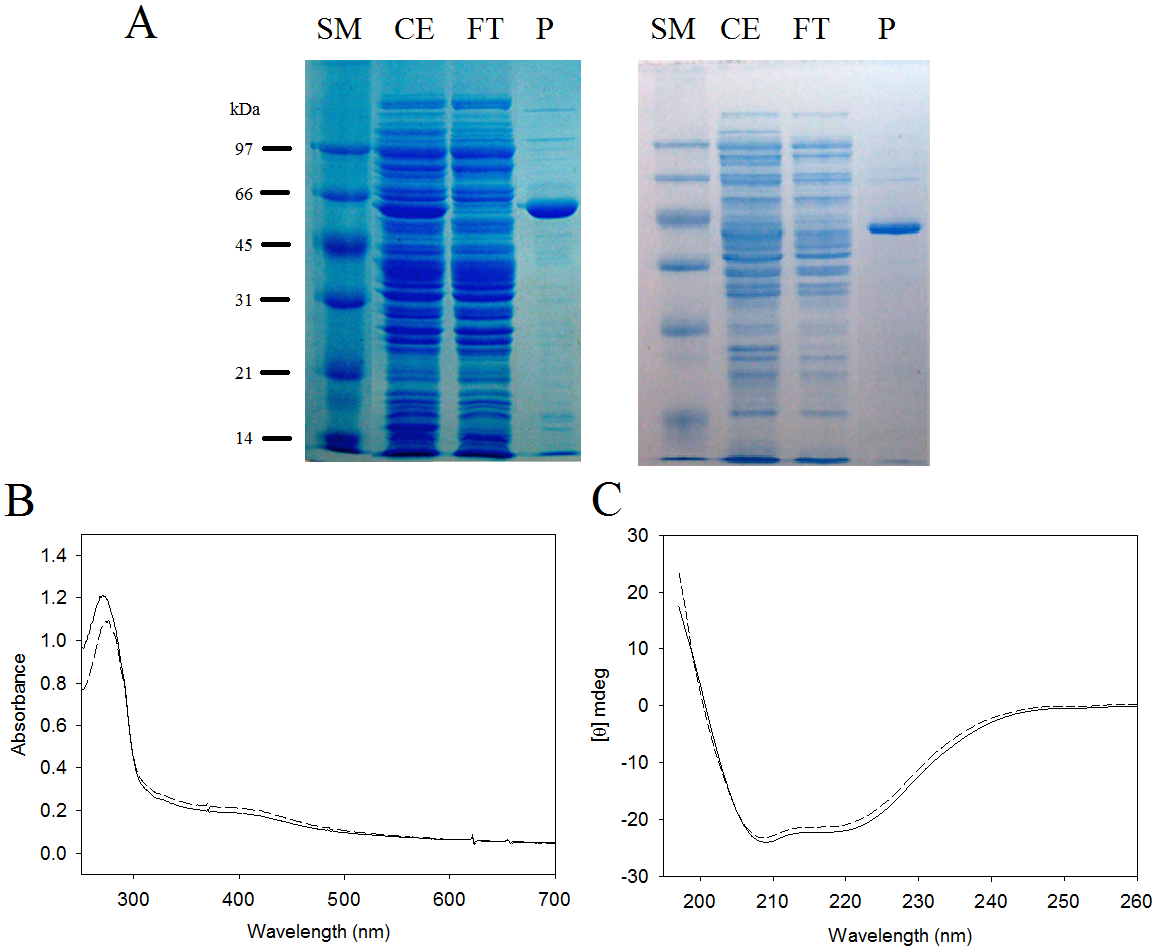


**Figure S3**. EPR spectra of GbHDR1-full (150 μM) in the presence of DT. The EPR-silent [Fe_4_S_4_]^2+^ cluster in the isolated protein developed typical ferredoxin-type rhombic signals upon reduction with DT. The reduced [Fe_4_S_4_]^+^ cluster also showed temperature-dependency, which is characteristic to the reduced cuboidal [Fe_4_S_4_]^+^ cluster. Spectra were recorded at 20K and 60K, microwave frequency 9.64 GHz, microwave power 0.94 mW, modulation frequency 100 kHz, modulation amplitude 10 G.


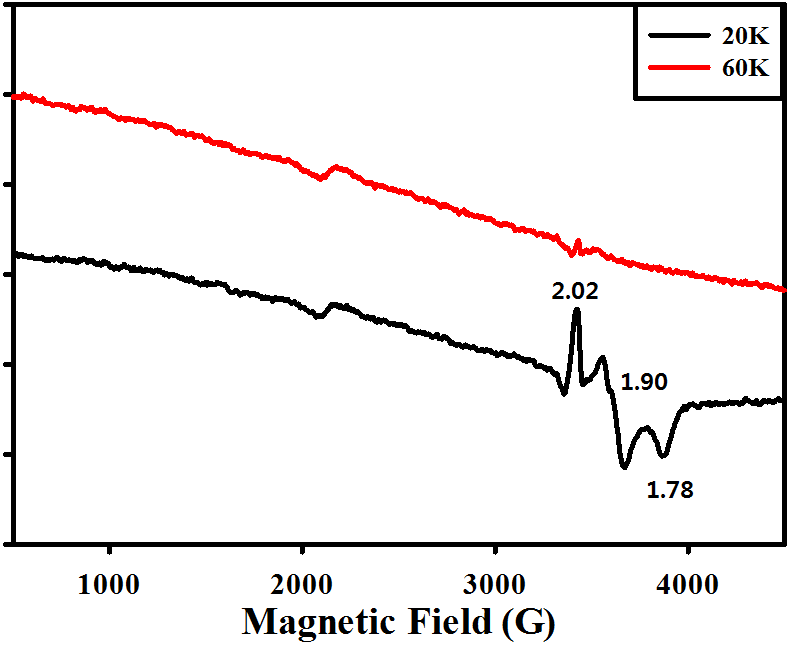


**Figure S4**. Michealis-Menten graphs of GbHDR1-truncated (solid line) and GbHDR1-full (broken line). Two-electron consumption was calculated as the consumption of one molecule of substrate.


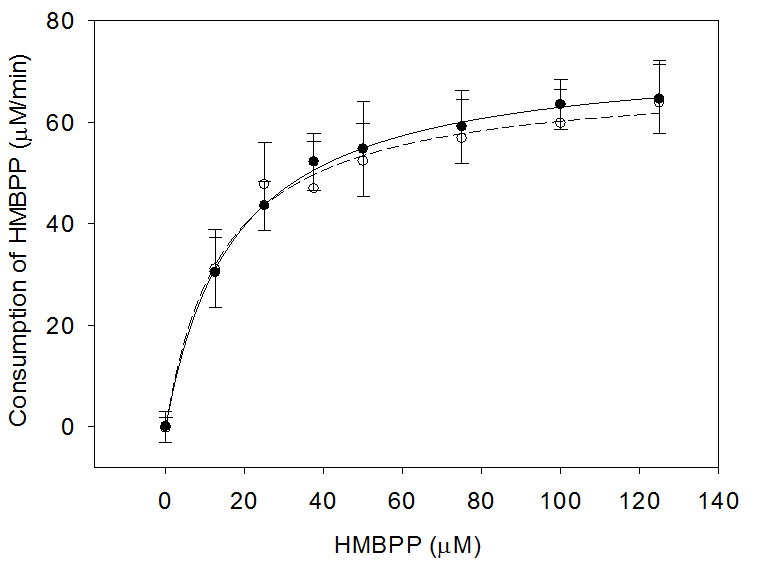
**Figure S5**. GC-MS chromatogram of the GbHDR1 reaction products (left) and the mass spectra of 3-methyl-3-buten-1-ol (A) and 3-methyl-2-buten-1-ol (B) in ethyl acetate extracts after dephosphorylation (right). Dotted line in chromatogram represents for the control data obtained without enzyme.


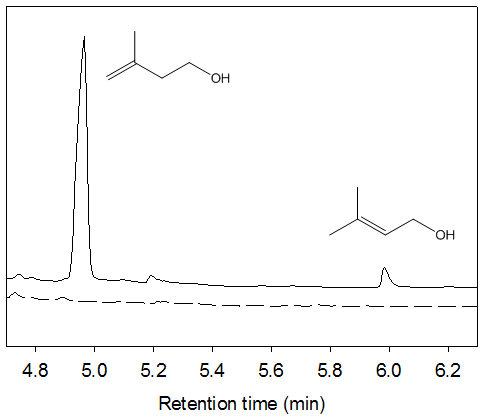

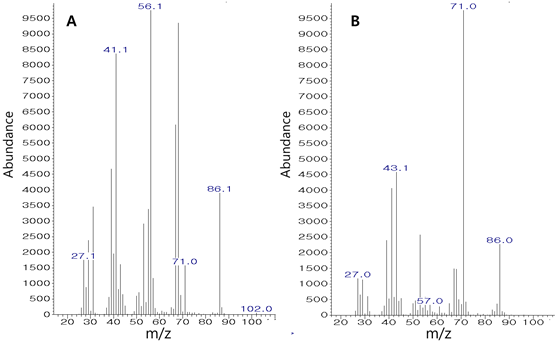


**Figure S6**. Amino acid sequences of GbHDR1-full (left) and GbHDR1-truncated (right).
